# Illinois State Dental Society

**Published:** 1868-06

**Authors:** 


### ARTICLE. VI.

### *Illinois State Dental Society.*

The Illinois State Dental Society met in the Senate Chamber in Springfield, May 12, at 11 o'clock a.m. Dr. G. H. Cushing, President ot the Society called the meeting to order. Dr. M. S. Dean acting as Secretary.

The following named members answered at roll call :

M. S. Dean, of Chicago ; S. Babcock, of Springfield ; G. H. Cushing, of Chicago J. H. Crouse, of Mt. Carroll ; O. Wilson, of Aurora ; E. H. Kilbourne, of Aurora ; Geo. P. Kingsley, of Freeport ; A. W. French, of Springfield ; H. J. Smith and H. H. Lewis, of Quincey.

After the proceedings of the previous meeting had been read, the following named gentlemen were elected members of the Society, and signed the constitution : T. D. Lamphiere, of Springfield ; G. S. Mills, Jerseyville ; G. V. Black, of Jacksonville; Charles Henry, of Jacksonville; G. W. Rivers, of West Pittsfield; C. O. Dean, of Quincey; F. E. Hansen, of Winchester; N. C. Hunting, of Lincoln; C. S. Smith, of Springfield.

The following named gentlemen were elected officers for the ensuing year:

*President*—E. H. Kilbourne, of Aurora.

*Vice President*—G. P. Kingsley, of Freeport.

*Secretary*—H. J. Smith, of Quincey.

*Treasurer*—A. W. French, of Springfield.

*Librarian*—M. S. Dean, of Chicago.

After some discussion, it was decided to hold the next meeting of the Society at Quincey.

The following named gentlemen were elected an executive committee for the ensuing year: H. N. Lewis, G. W. Rivers, O. C. Dean, J. N. Crouse and G. V Black.

After the election of the committee, Drs. C. K. Sawyer of Jacksonville, and J. M. Hunt of Lincoln, were unanimously elected members of the Association.

At this stage of the proceedings, Dr. E. H. Kilbourne, the newly elected President, took the Chair and thanked the Association for the honor conferred upon him in electing him President of the Association.

Dr. Cushing reported on the design of the society's seal, which was adopted.

Dr. Kilbourne then read a brief paper on the treatment of decayed six year molars, which was listened to with marked attentionr

SECOND DAY.

The Association met pursuant to adjournment. President Kilbourne presiding.

On motion of Dr. Miles, the rules were suspended for the election of members. Dr. S. L. Edwards, of Griggsville was proposed for membership, and the matter referred to the Executive Committee.

On motion, of Dr. Cushing, Dr. Edwards was invited to participate in the proceedings of the Association.

On motion Dr. H. J. McKellops and Dr. Porre, of St. Louis, were elected honorary members of this Association.

On motion of Dr. French, the discussion of the subject of the treatment of six-year molars was passed, and the next subject—" Plugging Pulp Cavities and Canals," was taken up for discussion."

Dr. M. S. Dean, of Chicago, read an able paper upon the subject. (See Editorial Matter.)

A discussion then ensued in which the following named gentlemen participated.

Dr. C. S. Smith advocated the use of cotton and kreosote for filling up pulp cavities and contended that kreosote forms with the animal matter of the tooth—insoluble, thus closing up the dental tubuli and rendering the canal impervious to the secretions.

Dr. Black uses gold in the form of a ribbon rolled on a broach and forces this into the canal to the apex of root.

Dr. Cushing cited several cases in which he extracted teeth and found gold in the form of wire protruding through the foramen, in one instance three eighths and in another one-eighth of an inch—uses cotton and kreosote for filling canals.

Dr. Rives thought that the majority of failures in fang filling was attributable to the imperfect manner in which the operations are performed. Uses gold generally. Does not regard cotton and kreosote entirely objectionable.

Dr. Judd of St. Louis, coincided with the essayist in the main—regards the indestructibility in a material for filling fangs absolutely necessary. Gold cannot be forced through the apicial opening of a fang, unless the cementum which closes the foramen be removed by absorption.

Dr. McKellops, of St. Louis, advocated the use of gold for filling roots, on account of its indestructibility; has removed teeth where the gold has become exposed by the absorption of the cementum from the apex of the fang.

Dr. Lewis asked whether a tooth in which a broach had been broken off in the foramen, be kept dry and filled would oxidize.

Dr. Judd answered that the cementum at the apex of the fang is permeable by the fluids, and that there was a probability of the secretions reaching the broach and causing oxidation.

On motion the meeting adjourned until three o'clock P. M.

AFTERNOON SESSION.

The Association met at three o'clock, pursuant to adjournment, President Kilbourne in the Chair. The executive committee reported in favor of the election of Dr. S. L. Edwards as a member of the Association. Report adopted and Dr. Edwards was declared a member, etc.

The chair announced the next subject for discussion to be "Receding of the Gums in Persons of Middle Age—Cause and Treatment."

Dr. Wilson read a very able and interesting paper upon this subject. A discussion then ensued upon the points referred to in the paper read.

Dr. Judd thought our researches upon the subject had failed to discover a satisfactory cause of the disease. It was not known whether the gums receded by absorption, or are washed away by an acid fluid secreted by the gums and acting upon the structure; thought the use of a stiff tooth brush, with constitutional remedies, would be the proper treatment in such cases.

Dr. Dean thought the absorption, when not caused by local irritation, is the result of defective nutrition.

Under the suspension of the rules Dr. Eames of St. Louis, presented a most interesting case of morbid anatomy—in which the nasal, palatal and anterior portion of the superior maxillary-bones were entirely destroyed—by Lupus—the disease was arrested and permanently cured by topical treatment.

Dr. Eames exhibited a most ingenious mechanical apparatus, which he is preparing to restore the lost part, which promises to be a success.

Dr. Freeman offered a paper on the "Observed effect of premature Extraction of temporary Teeth," in which he affirmed that the premature extraction of temporary teeth frequently produced irregularities and sometimes, as he thought, retarded the early coming of the permanent teeth.

Dr. Forbes thought the development of the maxillary bones did not depend on the presence of the teeth, that the blood vesseis that permeate the maxillary, supply the osseous material for its formation,—had not observed any ill effects from premature extraction of deciduous teeth. Dr. Cushing had not been able to discover any bad results from the premature extraction of the deciduous teeth ; he never removed them unless they became a source of irritation through disease. Dr. French regarded the presence of temporary teeth necessary for the developement of the jaw.

Dr. McKellops opposed the injudicious extraction of the temporary teeth, and only resorted to removal when the necessities of the case demanded it.

Dr. Cushing read a paper on "Facial Neuralgia," which called forth considerable discussion, in which many members joined.

The Association then adjourned until 8 o'clock P. M.

EVENING SESSION.

The Association met at 8 o'clock, Wednesday evening, pursuant to adjournment, Dr. Kilbourne presiding.

The proceedings of the morning session were read and approved.

Dr. Crouse moved that the election of delegates to the American Dental Association, be the first business in order for the morning session. Carried.

The subject of "Facial Neuralgia" was passed and "Anæsthesia" was announced by the Chair as next in order.

Dr. Cushing, of Chicago, read a paper upon the duties of the profession in the use of anæsthetics, in which he briefly reviewed the merits of the two principal agents now in use, viz.: chloroform and nitrous oxide, as to their safety and desirability, citing chiefly from Professor Watt, of Cincinnati, his experiments and deductions, and drawing from the evidence the conclusion that much greater caution was necessary in the preparation of nitrous oxide than probably was generally used ; and that its preparation and administration were undertaken by a vast number of incompetent and irresponsible parties, but establishing upon such evidence as was attainable, the fact of the much greater safety of nitrous oxide, as well as its preferable qualities over chloroform in many other respects, and concluding with an earnest appeal for a full investigation of the subject in order to secure simplicity and certainty in its preparation; to establish, if possible, a standard by which it may be easily tested, and by every safe-guard which can be thrown around it,—to place it upon the most assured ground of safety and reliability, under the conviction that eventually it would prove to be the greatest boon to suffering humanity which a merciful Providence could vouchsafe.

Considerable discussion ensued upon the question as to the merits of different anæsthetic agents, in which Drs. Wilson. Judd, Freeman and others participated.

On motion the Society adjourned until Thursday morning at 10 o'clock.

THIRD DAY.

The State Dental Association met yesterday morning at 10 o'clock, pursuant to adjournment, President Kilbourn presiding.

The reading of the proceedings of the previous meeting were dispensed with.

The first order of business being the election of delegates to the American Dental Association, which meets at Niagara Falls, on the last Tuesday in July next, the Chairman appointed Drs. French, Rives and Henry, a committee to nominate delegates.

Dr. Cushing read a letter from Dr. T. F. Woodbridge, of Mendota, Illinois. Letter placed on file.

Dr. Cushing moved to take up the subject of "Facial Neuralgia," in order to give Dr. Stoddard Smith, an opportunity to bring before the association two cases that came under his observation, where the disease was caused by ossification of the pulp. Adopted.

Dr. Smith then presented the subject for the consideration of the association.

Dr. Forbes asked whether the hygenic habit stimulated the deposit of ossific matter, or whether it was morbid deposition.

Dr. Cushing thought it a morbid condition.

After some discussion of the subject, on motion of Dr. Smith the subject was passed, and Anæsthetics was taken up to give Dr. Wilson an opportunity to exhibit an "improved inhaler" for chloroform, and other vapors and gases. He considered the easy working of the valves of the instrument as very important in the process of inhaling. The subject was then passed; after which Dr. Black offered the following resolution which was adopted:

*Resolved*, That a committee of three be appointed to prepare an address to the people on the subject of the importance of the treatment of the "six-year molars," and that such address be presented to the next meeting of this body for their consideration.

On motion of Dr. Kingsley, Drs. Kilbourne, Black and Cushing were appointed said committee.

The committee on the nomination of delegates to the American Dental Association, reported the following named gentlemen as delegates:

Charles Henry, Jacksonville; G. S. Miles, Jerseyville; O. Ames, Kankakee; C. S. Smith, Springfield; F. E. Hanson, Winchester; H. J. Smith, Quincy; S. L. Edwards, Griggsville; J. D. Kilbourne, Chicago; O. Wilson, Aurora; A. W. Freeman, Chicago; J. Crouse, Mount Carroll; George P. Kingsley, Freeport; G. V. Black, Jacksonville.

The report was unanimously adopted.

Dr. Kingsley moved that the delegates be empowered to fill all vacancies which may occur in the delegation. Carried.

The regular order of business was taken up. The subject of "Mechanical Dentistry" was announced by the chair as the next in order. Dr. Eames then addressed the Association upon the subject, giving a detailed statement of his method of taking impressions of the mouth, and of different materials used as a base for mounting artificial teeth. After discussing the subject for a short time, the matter was passed.

Dr. Kilbourne, President of the Association, then offered the following resolutions:

*Resolved*, That the Illinois State Dental Society hereby endorse in full the action of the American Association at its last session at Cincinnati, in relation to the report of committee appointed by said Association to examine the credentials of the delegates from the so called St. Louis Dental College which, report justly declares, has no legal existences and that a Diploma from such an Institution would be utterly worthless.

*Resolved*, That this so-called Dental College, so far from being an honorable institution of learning, is only a shop for manufacturing diplomas which are hawked about as any other wares or merchandise are hawked about by hucksters, and that any one engaged in such nefarious practice is unfit for fellowship with honorable men.

*Resolved*, That as dentists, seeking the highest good of our noble profession, we will use all honorable means to build up our Dental Colleges that are laboring to advance the interests of dental science.

The resolutions were adopted.

On motion, Dr. M. S. Dean, of Chicago, was selected to address the convention at its next annual meeting.

Dr. Miles offered a resolution thanking the Secretary of State, Hon. Sharon Tyndale, for the use of the Senate Chamber, which was unanimously adopted.

On motion the convention adjourned to meet at Quincey on the second Tuesday in May, 1869.

---

